# High coverage but low quality of maternal and newborn health services in the coverage cascade: who is benefitted and left behind in accessing better quality health services in Nepal?

**DOI:** 10.1186/s12978-022-01465-z

**Published:** 2022-07-19

**Authors:** Resham B. Khatri, Jo Durham, Rajendra Karkee, Yibeltal Assefa

**Affiliations:** 1 School of Public Health, Faculty of Medicine, The University of Queensland, Brisbane, Australia; 2 Health Social Science and Development Research Institute, Kathmandu, Nepal; 3 School of Public Health and Social Work, Queensland University of Technology, Brisbane, Australia; 4 School of Public Health and Community Medicine, BP Koirala Institute of Health Sciences, Dharan, Nepal

**Keywords:** Antenatal care, Institutional delivery, Postnatal care, Coverage cascade, Determinants, Quality, Maternal and newborn health, Nepal

## Abstract

**Background:**

Antenatal care (ANC) visits, institutional delivery, and postnatal care (PNC) visits are vital to improve the health of mothers and newborns. Despite improved access to these routine maternal and newborn health (MNH) services in Nepal, little is known about the cascade of health service coverage, particularly contact coverage, intervention-specific coverage, and quality-adjusted coverage of MNH services. This study examined the cascade of MNH services coverage, as well as social determinants associated with uptake of quality MNH services in Nepal.

**Methods:**

We conducted a secondary analysis of data derived from the *Nepal Demographic and Health Survey* (NDHS) *2016*, taking 1978 women aged 15–49 years who had a live birth in the 2 years preceding the survey. Three outcome variables were (i) four or more (4^+^) ANC visits, (ii) institutional delivery, and (iii) first PNC visit for mothers and newborns within 48 h of childbirth. We applied a cascade of health services coverage, including contact coverage, intervention-specific and quality-adjusted coverage, using a list of specific intervention components for each outcome variable. Several social determinants of health were included as independent variables to identify determinants of uptake of quality MNH services. We generated a quality score for each outcome variable and dichotomised the scores into two categories of “poor” and “optimal” quality, considering > 0.8 as a cut-off point. Binomial logistic regression was conducted and odds ratios (OR) were reported with 95% confidence intervals (CIs) at the significance level of p < 0.05 (two-tailed).

**Results:**

Contact coverage was higher than intervention-specific coverage and quality-adjusted coverage across all MNH services. Women with advantaged ethnicities or who had access to bank accounts had higher odds of receiving optimal quality MNH services, while women who speak the Maithili language and who had high birth order (≥ 4) had lower odds of receiving optimal quality ANC services. Women who received better quality ANC services had higher odds of receiving optimal quality institutional delivery. Women received poor quality PNC services if they were from remote provinces, had higher birth order and perceived problems when not having access to female providers.

**Conclusions:**

Women experiencing ethnic and social disadvantages, and from remote provinces received poor quality MNH services. The quality-adjusted coverage can be estimated using household survey data, such as demographic and health surveys, especially in countries with limited routine data. Policies and programs should focus on increasing quality of MNH services and targeting disadvantaged populations and those living in remote areas. Ensuring access to female health providers and improving the quality of earlier maternity visits could improve the quality of health care during the pregnancy-delivery-postnatal period.

**Supplementary Information:**

The online version contains supplementary material available at 10.1186/s12978-022-01465-z.

## Plain Language summary

Utilisation of essential maternity and newborn health (MNH) interventions during pregnancy, childbirth, and the postnatal period can improve the health of mothers and newborns. There are increasing access trends for routine MNH visits in Nepal, such as at least four (4^+^) antenatal care (ANC) visits, institutional delivery, and postnatal care (PNC) visits. Little is known, however, about the utilisation of recommended MNH interventions, or who is benefitting or being left behind in relation to better quality MNH services. This study examined different types of health service coverage cascades across routine MNH services and associated determinants using *Nepal Demographic and Health Survey 2016* data. A total of 1978 women aged 15–49 years who had live-births 2 years preceding the survey were included in the analysis. Despite improved access to routine visits during the pregnancy-postnatal period, some women had not received all recommended interventions, and had low-quality-adjusted coverage of MNH services. Women with advantaged ethnicities or financial assets received optimal quality MNH services, while Maithili-speaking women or those with high birth order received poor quality ANC services. Women from Karnali province or women who preferred to be seen by a female healthcare provider experienced poor quality PNC service. Demographic and health survey data can be used to assess the population-level coverage of quality health services. Policy and program efforts require better quality MNH services that employ female health providers.

**Supplementary Information:**

The online version contains supplementary material available at 10.1186/s12978-022-01465-z.

## Introduction

Maternal and neonatal mortality has declined in recent decades; however, discrepancies in pregnancy outcomes remain, with most deaths concentrated in the low- and lower-middle-income (LMICs) countries in Sub-Saharan Africa and South and South-East Asia [1]. Most of these deaths are preventable through improved access to quality essential antenatal, intrapartum, and postnatal interventions. According to the World Health Organization (WHO), every pregnant woman (and newborn) should receive these essential interventions [2, 3]. During pregnancy, women should receive at least four antenatal care (ANC) visits encompassing antenatal interventions (e.g., iron supplementation, tetanus toxoid immunisation) and be screened for high-risk pregnancy [4]. Intrapartum care interventions include childbirth assisted by skilled birth attendants, and all women and newborns should receive intrapartum and immediate newborn care interventions [5]. Postnatal care (PNC) interventions cover the examination of mother and their babies for any infections, advice for hygiene and sanitation, and advice for nutrition and family planning [6] and every woman (and newborn) should undertake at least three PNC visits during the first week of childbirth [7].

Although access to routine maternity and newborn health (MNH) in many LMICs has increased over recent decades [8, 9], the reduction of the Maternal Mortality Ratio (MMR) and the Neonatal Mortality Rate (NMR) remains slow [10]. One reason for this slow reduction is poor uptake of essential MNH services [3]. Another reason is deficiencies in the quality of care, and the fact that, even when women access health facilities, they may not receive all recommended interventions [11]. Improving MNH outcomes, therefore, requires examination of the actual receipt and quality of essential interventions, levels of coverage, and determinants associated with accessing quality health services [12, 13].

*The Lancet Commission of High-Quality Health System* report has shown that two-thirds of mortality is attributed to poor quality of care [14]. The same report noted improved health system quality is a prerequisite for better-quality services, including a competent health workforce, equipment, monitoring, supervision mechanisms, and an enabling policy and governance environment [14, 15]. The quality of MNH care as a determinant of outcomes is also emphasised by Sustainable Development Goal Three (SDG 3) and its associated policies and programs [15–18].

In 2019, World Health Organisation and United Nations Children’s Fund convened a group of experts or the Effective Coverage Think Tank Group to recommend a framework for measurement of effective coverage of health services [16]. The group recommended a measurement framework on coverage cascade based on the generic cascade proposed by Amouzou and colleagues [3], which builds on the Tanahashi framework [19] for evaluating health-service coverage and allows for population-level assessment of health services along the maternal and child health continuum of care [16]. The health service coverage cascade provides a tool for assessing health-system performance across the sequence of interactions between patients and the health system at different stages. The coverage cascade includes service contact coverage (the proportion of the population in need who come into contact with the (relevant) health service); input-adjusted coverage (refers to who come into contact with a health service that is ready to provide care); intervention coverage (refers who come into contact with a service that is ready and that receives the service) [16]. Similarly, other stages of on coverage cascade include quality-adjusted coverage (who comes into contact with a service that is ready and that receives the service according to quality-of-care standards); user adherence-adjusted coverage (refers to who receives the service according to quality-of-care standards and that adheres to provider instructions); and outcome-adjusted coverage (refers to who receives the service according to quality-of-care standards, adheres to provider instructions, and has the expected health outcome) [16]. The quality-adjusted coverage of health services incorporates the population in need of health services, the proportion of health service contact, and the composite coverage of intervention/procedures of health services [2, 16, 20–22]. In addition, it also includes the frequency and adequacy of services provided by skilled health workers and service uptake (of essential interventions) at health facilities [3, 15, 23], the delivery procedure, and uptake of technical interventions, including respectful care [24, 25], providing a proxy measure of the quality of health services and health system performance [26, 27].

In Nepal, despite significant progress, maternal and newborn health continues to be a major public health concern. In Nepal, the MMR is high (259 per 100,000 live births), and so is the NMR (21 per 1000 live births) [28]. Further, declines in the MMR and NMR have slowed substantially compared to the increase in utilisation of routine MNH visits over the past two decades. For instance, from 2006 to 2016, institutional delivery increased from 18 to 59%, while the MMR only decreased from 281 per 100,000 live births to 259 per 100,000 in the same period [29]. Additionally, while coverage of routine MNH visits has increased, rates of improvement are higher among more privileged groups compared to their disadvantaged counterparts, suggesting equity gaps exist [30, 31]. Evidence suggests the NMR and MMR are highest among disadvantaged groups, who have poorer coverage of routine MNH visits and receive poorer quality of care during those routine visits [29]. This means disadvantaged women have poor access to recommended interventions and/or the health system is inefficient in delivering those interventions during routine MNH visits.

Nepal has achieved considerable success in increasing nationwide coverage and compliance with iron and folic acid supplementation during pregnancy [32]. Antenatal micronutrient supplementation and educational interventions have successfully improved maternal and neonatal outcomes [33]. Some health facility-level factors have contributed to better quality MNH services. For instance, women received good services at health facilities with better readiness, or services provided by nursing staff or supervised health facilities/health workers [34]. In addition to service provision, users’ experience of health care, including respectful care of mothers and newborns around the time of birth, is crucial for better quality care [35]. The COVID-19 pandemic has had differential effects on maternity services, with changes varying according to volume of births per hospital, where smaller facilities have increased volumes of use of maternity care [36]. However, there is little evidence on social determinants associated with uptake of better quality MNH services in Nepal, even prior to COVID-19.

Although studies have examined contact coverage of routine MNH services [37, 38] and perceived quality of care [39, 40], there has been little research conducted on the population-level quality of care of quality-adjusted coverage routine MNH services [41]. This study examines (i) the cascade of health service coverage (gaps in contact coverage, intervention-specific coverage, and quality-adjusted coverage) at the population level, (ii) social determinants associated with access to quality of routine MNH services. The study provides recommendations to address these gaps that could inform programs and policies targeting disadvantaged groups. Additionally, the study identifies modifiable factors of poor quality of care that can inform the design of evidence-based interventions aiming to reduce equity gaps in access to quality health services. In doing so, the study contributes to decreasing the MMR and NMR in Nepal and similar settings.

### Nepal’s policy context for quality maternal and newborn care services

Current MNH policies of Nepal emphasise quality health care; for instance, the *National Health Policy 2019* [42], and the *Nepal Health Sector Strategy (2016–2021)* focuses on quality health care, equity, multisectoral action and heath sector reform [43]. *The Nepal Safe Motherhood and Newborn Health Programme Roadmap 2030* [44], *Nepal Newborn Action Plan (2015–2035)* [45], and *Skilled Birth Attendant (SBA) Strategy (2020–2025)* [46] also highlight the importance of the quality of MNH services in improving outcomes [47]. Quality improvement initiatives, such as the Maternal and Perinatal Death Review, minimum standard of health facility assessment tools, routine clinical monitoring, and supportive onsite supervision visits are stated in policy documents and guidelines as a means for improving quality of care but are not properly functioning as envisioned [48]. Existing routine health management information systems (HMIS) [48] and other monitoring mechanisms or surveys measure the quality of health services in Nepal [49, 50].

## Methods

### Study context and setting

Nepal is a multiethnic, multilinguistic, landlocked country between China in the north and the rest (south, east, and west) by India. One-third of the population live below the multidimensional poverty level, with an average per capita purchasing power of 1061 USD. Nearly two-thirds of the economy relies on foreign remittance and the agricultural sector [51].

**Fig. 1:**
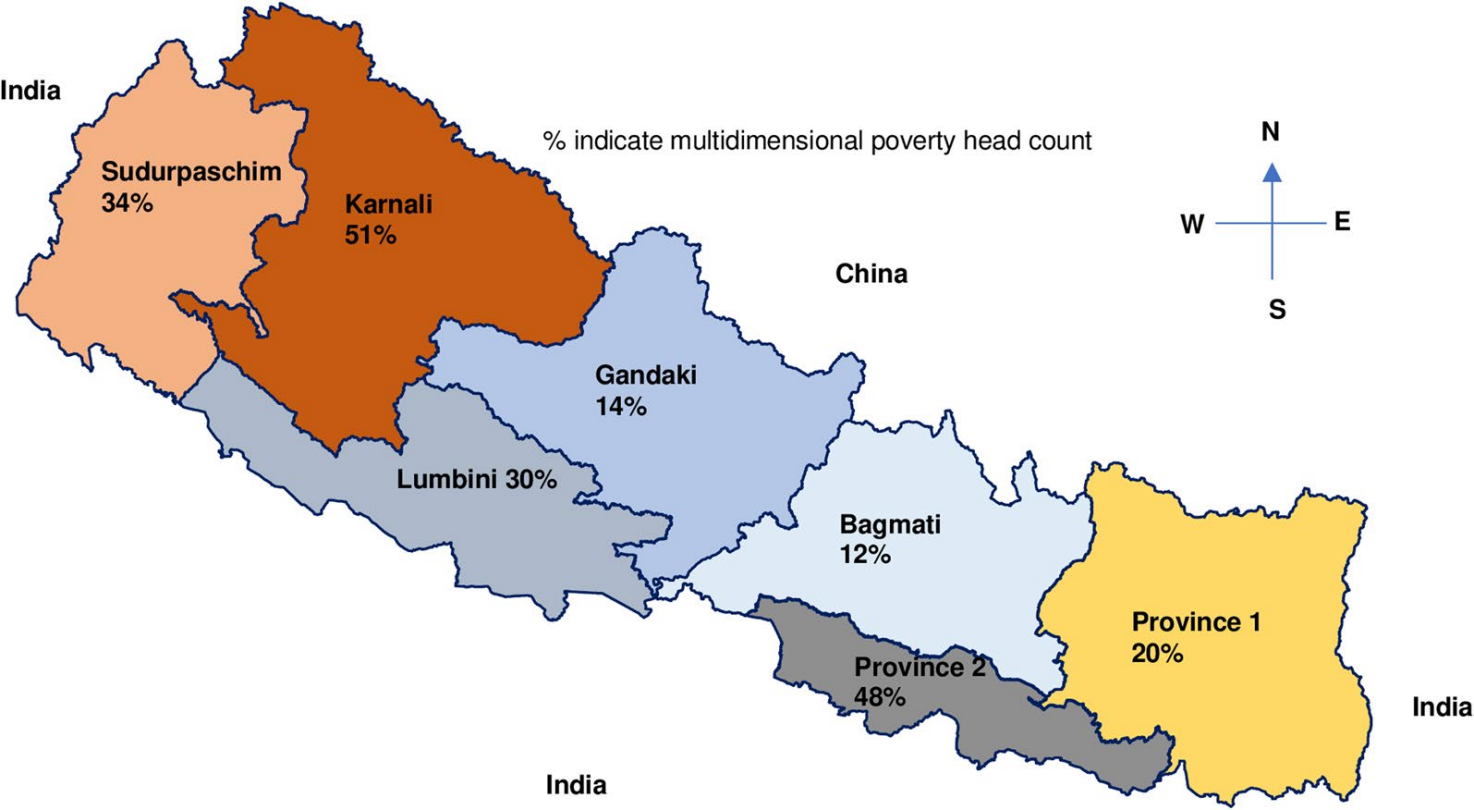
Map of Nepal showing province specific poverty level (Adapted from [51])

Nepal has a federal governance system, with seven provincial and 753 local municipal governments (Fig. 1). Madhesh province lies in the plain region bordered by India. About half of the population speaks a language other than Nepali (Maithali) in this province. Karnali province is the remotest province, bordered by China, and is covered by hills and mountains. Karnali and Madhesh provinces have high poverty rates [52] (Fig. 1).

### Study design and sampling

**Fig. 2:**
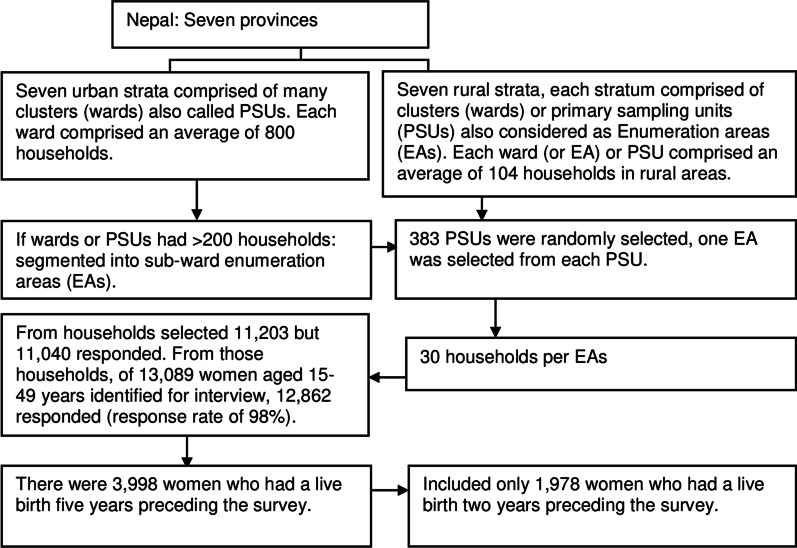
Summary of sampling design used in the NDHS 2016

This was a cross-sectional study based on the further analysis of data derived from the *Nepal Demographic and Health Survey* (NDHS) *2016* [29]. The NDHS 2016 was a nationally representative cross-sectional household survey based on generic designs of the Demographic and Health Survey program. The NDHS 2016 represents the fifth round of the NDHS (which is conducted every 5 years). Demographic and health surveys are conducted to assess health services performance, especially reproductive, newborn, child health and nutrition, in more than 90 LMICs [29]. The present study extracted data from individual woman’s records from the 2016 NDHS. A more detailed sampling method is described in the NDHS 2016 report [29, 53], and the sample size included in this study is detailed in Fig. 2. In total, 1978 women aged 15–49 years who had had a live birth in the 2 years preceding the survey were included in the analysis. In addition, we included information on ANC, childbirth, and PNC interventions to calculate the different cascades of coverage.

### Conceptual framework for the selection of study variables and analysis

**Fig. 3:**
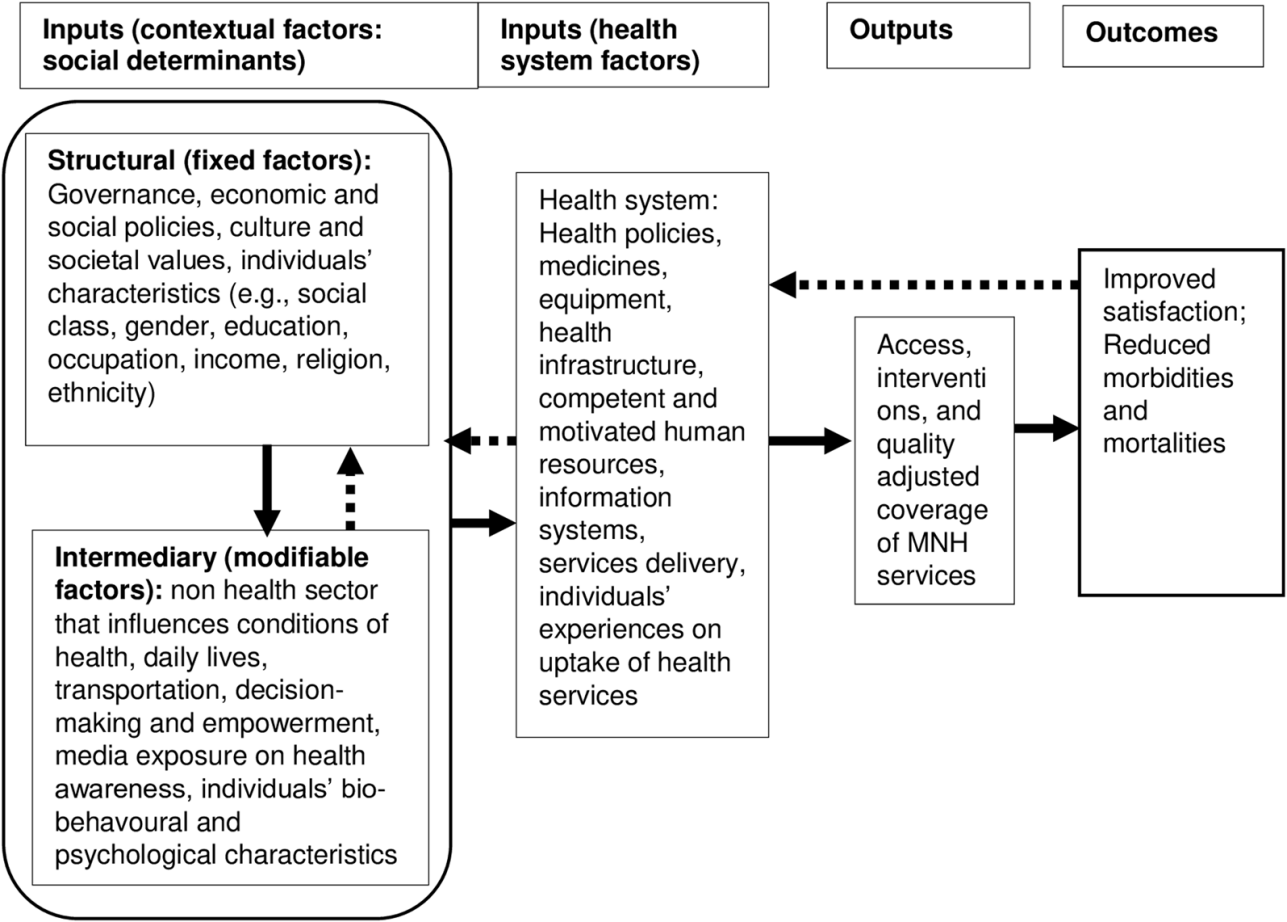
A conceptual framework adapted and modified from the WHO’s Commission of Social Determinants of Health [56]

Based on the review of previous conceptual frameworks, including the WHO’s social determinants of health [54–56], we developed a guiding conceptual framework [34] for this study (Fig. 3). This conceptual framework comprises inputs that include several correlates under structural, intermediary and health system domains. Structural variables are fixed, difficult to modify, and need political interventions, while intermediary variables include modifiable factors and cover non-health sector factors. The health system factors are related to the health sector and supply of health services. These correlates can operate at the system, institutional, and individual levels that influence the utilisation of quality MNH services [34].

Independent variables included characteristics of women and their healthcare experience (Additional file 1: Table S1). Based on available information in the NDHS 2016 data and guided by the conceptual framework (Fig. 3), we selected several independent variables [57]. The variables under the structural domain were ethnicity, wealth status, education, religion, maternal occupation, and decision-making for at least three areas (healthcare, purchasing, and movement). Ethnicity, wealth status and maternal education were further categorised using previous studies and government reports. In the NDHS 2016, wealth quintiles were constructed using principal component analysis based on more than 40-asset items owned by households [57]. These wealth quintiles were merged into two groups: the lowest two quintiles as Poor (lower 40%) and the upper three quintiles as Rich (upper 60%) [53].

For reporting by its routine health information system, Nepal categorised 123 ethnicities into six broader categories: (i) Dalits (untouchable), (ii) disadvantaged indigenous, (iii) disadvantaged non-Dalit Terai caste groups, (iv) religious minorities (Muslims), (v) relatively advantaged indigenous, and (vi) upper-caste groups [58]. These broader ethnic groups were merged into two groups according to their comparative privileges: disadvantaged ethnicities (includes Dalit, Muslims, and non-Dalit Terai caste, disadvantaged Janajatis) and advantaged ethnicities (includes Brahmin or Chhetri, advantaged Janajatis) [53].

Maternal education was categorised as illiterate (who cannot read and write), primary (who can read and write and up to grade 8 level), and secondary and higher (who have grade 8 and higher-level education) [53]. Independent variables under the intermediary domain were age of mothers, first language, residence, provinces, region, birth order, sex of the child, access to a bank account, media exposure, distance to health facilities as a perceived problem, and intended birth of index child (last child) child. Health system factors included the (perceived) problem of not having female healthcare providers, awareness of health mothers’ groups, mode of delivery, and quality of four or more ANC visits and institutional delivery. This study examined the health service cascade and social determinants of health on the utilisation of quality MNH services using three outcome variables: four or more (4^+^) ANC visits [59], institutional delivery and at least one PNC visit by mothers and newborns within 48 h of childbirth.

### Framework of health service coverage cascade

**Fig. 4:**
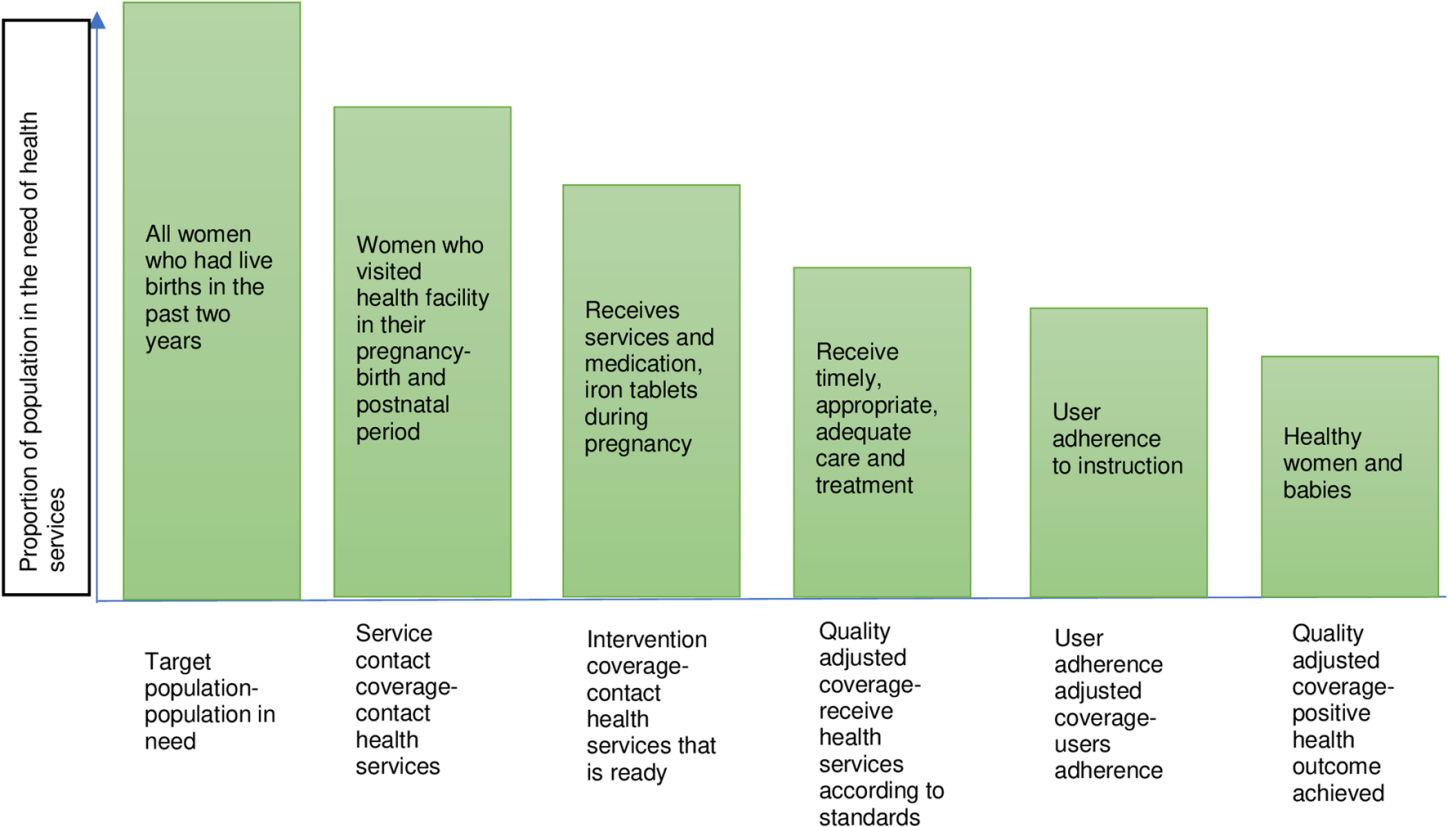
Health services coverage cascade for MNH services (Adapted and modified from Marsh and colleagues [16])

We applied the health service coverage cascade adapting from Marsh and colleagues to quantify the use of services at the different conditional stages [16, 20] (Fig. 4).

Table 1 shows the different levels of the coverage cascade as defined for this study [16]. In brief, the health service coverage cascade conceptualises the coverage as (i) Service contact or contact coverage, which is the proportion of the target population who visit a health facility for care; (ii) Intervention-specific coverage, which is the proportion of the target population who receive a needed health intervention (e.g., uptake of tetanus toxoid immunisation in ANC visit); and (iii) quality-adjusted coverage, which is the proportion who receive service according to recommended standards covering multiple dimensions of care such as timely, frequent and adequate care provided skilled health providers.

**Table 1:** Cascade of health service coverage in pregnancy, childbirth, and postnatal period

Services	Target population	Service contact	Intervention-specific coverage	Quality-adjusted coverage
Antenatal care visit	Women aged 15*–*49 years who had a live birth in the 2 years preceding the survey	Target population who had at least four ANC visits from skilled providers	Among women who had at least four ANC visits for their most recent birth, the coverage of key components of ANC (see Additional file 1: Table S2)	Received quality-adjusted coverage of 4^+^ ANC visits = average score of quality of 4^+^ ANC visits (Q) × proportion of 4^+^ ANC visits
Institutional delivery	Women aged 15*–*49 years who had a live birth in the 2 years preceding the survey	Target population who delivered in a health facility (institutional delivery)	Among women who delivered in a health facility, the coverage of key components of intrapartum care (see Additional file 1: Table S2)	Received quality-adjusted coverage of ID = average score of quality of ID (Q) × proportion of ID
Postnatal care visit	Women aged 15*–*49 years who had a live birth in the 2 years preceding the survey	Mother and newborn who received PNC visit within 48 h of childbirth	Among mothers and newborns who received PNC within 48 h, the coverage of key PNC components (see Additional file 1: Table S2)	Received quality-adjusted coverage of PNC visit = average score of quality of PNC visit (Q) × proportion of PNC visit

*EC *effective coverage (%), *Q* = average quality score of all interventions (ranges 0 to 1), *U *utilisation of contact coverage (range 0% to 100%), *ID *institutional delivery

Based on the WHO’s *Standard for Maternal and Newborn Care* [60] and Nepal’s national medical standards for maternal and newborn care [61], we identified several antenatal, delivery, and postnatal care interventions from the NDHS 2016 dataset (Additional file 1: Table S2). For quality of any MNH visit, women should receive sets of interventions by skilled providers, and timely, adequate and appropriate content of care during the pregnancy [5], childbirth [62] and postnatal period [63]. We selected 16 ANC interventions (e.g., iron supplementation, advice for skilled birth attendants assisted delivery, tetanus toxoid immunisation), eight interventions (e.g., assisted delivery, immediate newborn care) for institutional delivery; and 11 PNC interventions (e.g., observed danger signs of newborns, counselling breast feeding) for mothers and newborns (Additional file 1: Table S2). To calculate the average quality score, first, we estimated individual quality scores based on intervention-specific coverage. For example, the quality score for a woman who completed 4^+^ ANC visits was calculated based on interventions received by a woman from the list of recommended interventions (if a woman received 10 items during 4^+^ ANC visits of 16 items, her average quality score would be (= 10/16 = 0.625). If a woman did not receive any interventions, her score would be 0 (0/10 = 0), and if she received all items, her quality score would be 1 (16/16 = 1) [2, 18]. Thus, the population-level average quality score for the 4^+^ ANC visits would be the mean of the individual quality score (> 0 to 1). We calculated the quality score of 4 + ANC visits, institutional delivery, and PNC visits using a similar procedure.

### Social determinants of access to quality MNH services

Unlike the coverage cascade of MNH services at the population level, quality MNH visits were identified among those utilised routine MNH visits. Out of 1978 women, 1401 completed four or more (4^+^) ANC visits, 1270 gave birth at health facilities, and 999 completed at least one PNC visit for mothers and newborns within 48 h of childbirth. Three outcome variables for the regression analysis included quality of (i) 4^+^ ANC visits, (ii) institutional delivery, and (iii) PNC visits.

For the logistic regression analysis, a quality score of each service of each woman who received particular service was dichotomised. Although there is no gold standard of cut-off points for categorising poor or optimal quality care, this study took reference cut-off points of 0.80 (out of 1:00); taking reference of studies undertaken in Kenya [64] and Nepal [65]. Quality score of 1:00 (100%) is the perfect good quality. Women score with > 0.8 (received 80% of interventions) means women received optimal quality MNH services. Previous studies considered 0.75 (out of 1:00) as cut off point to dichotomise the good and poor quality MNH services. The higher the scores indicate optimal quality; therefore, considered the cut-off point higher than previous literature. Among those who completed the recommended MNH visits, the quality score was dichotomised into optimal quality if the score was > 0.8 = 1, and poor quality if the score was score > 0 and ≤ 0.8 = 0. The binary outcomes of each variable were coded as: 4^+^ ANC visits: 1 = optimal quality; 0 = poor quality; Institutional delivery: 1 = optimal quality; 0 = poor quality; PNC visit: 1 = optimal quality; 0 = poor quality.

### Statistical analyses

Descriptive analysis (frequency and proportion) was conducted on the coverage cascades (contact, intervention-specific and quality-adjusted) for all MNH services among all eligible populations (N = 1978). Contrarily, in the regression analysis, we considered women who received specific health services (4^+^ ANC visits; institutional delivery, and the first PNC visit) and identified determinants associated with access to quality MNH services. Multivariable binomial logistic regression analyses were conducted for each outcome variable (4^+^ ANC visits; institutional delivery, and the first PNC visit) to identify correlates of quality of MNH service using odds ratios (OR) with 95% confidence intervals (CIs). Before running the final regression model, multicollinearity was checked: independent variables having variation inflation factor (VIF) ≥ 3 were excluded [66, 67]. Next, backward elimination multivariable logistic regression analyses were conducted [68]. First, a full multivariable regression model was run; then p-values for each independent variable were estimated and statistically non-significant variables identified. This procedure was repeated until no insignificant independent variable was left at p < 0.2 [69]. The adjusted odds ratios (aOR) with 95% CIs for all independent variables retaining p < 0.05 were reported. The goodness of fit test was conducted using the Hosmer Lemeshow test (non-significant results (p > 0.05) indicated an adequate fit) [70]. Statistical significance level was set at p < 0.05 (two-tailed) to identify the social determinants associated with the outcome variable. All reported estimates were weighted (unless otherwise indicated). All analyses were conducted using the ‘svy’ command function and considering the clustering effect in Stata 14.0 (Stata Corp, 2015).

## Results

### Background characteristics of women

Details of background characteristics of women are presented in Additional file 1: Table S3. In brief, among 1978 women, 42% were from households in the lowest two wealth quintiles. More than two-thirds (69%) of women were from disadvantaged ethnic groups, mostly Madhesi, Janajatis and Dalits. Nearly two in five women (42%) were native Nepali speakers (the national language). More than half (55%) of women were from the Terai (Plain) Region. One in four women (26%) were from province two, whereas one in 20 women (6%) were from Karnali province. About half (46%) of the women were from urban areas. Two-thirds (67%) of the women had no decision-making authority (or empowerment) in relation to access in health-seeking, buying something (financial empowerment) or meeting with relatives (movement authority). Nearly one-third (29%) of women reported experiencing domestic violence (e.g., beating when food burnt or women went out without asking their husbands). Four in five (79.7%) women were aged 20–34 years, and approximately 69% of women did not have a bank account. Three in five women felt the distance to an HF was a challenge in accessing health services. Further, nearly 72% of women perceived it as challenging to access care when there was no available female health care provider. Over two-thirds (68%) of women had no awareness of the availability of a health mothers’ group in their community. One in 10 mothers delivered babies via caesarean section (C-section).

### Health service coverage cascade of MNH services

**Fig. 5:**
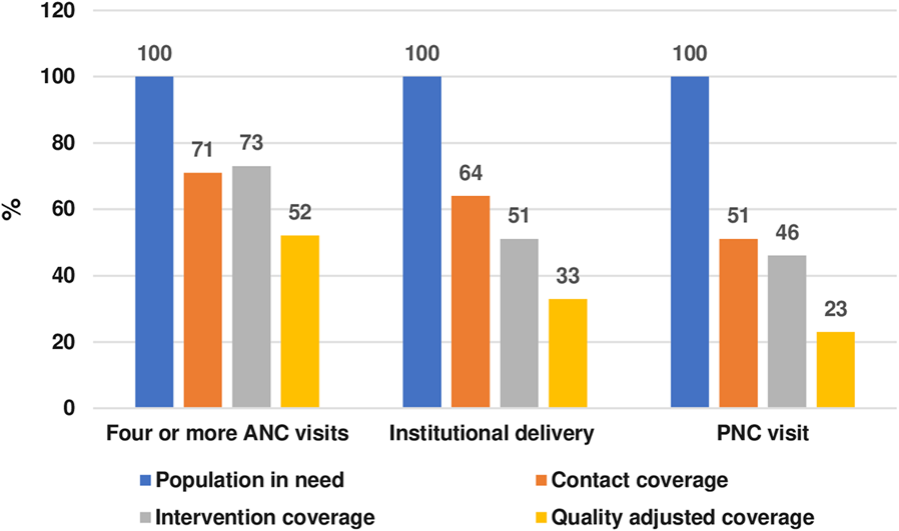
Health service coverage cascade MNH visits in Nepal

Figure 5 shows the results across the health serivce coverage cascade (N = 1978). Table 2 presents the contact coverage and intervention-specific coverage of each routine MNH visit. For the contact coverage among 1978 women who had a live birth in the past 2 years, 71% received four ANC visits; 64% were delivered at health institutions, and 51% of mothers and newborns received at least one PNC visit within 48 h of childbirth. The intervention-specific coverage for 4 + ANC visits was higher than the contact coverage (73%), as women received some ANC interventions from the female community health volunteers (volunteer community health workers) (e.g., iron, tetanus toxoid vaccine from immunisaiton outreach clinic). However, the intervention-specific coverage for institutional delivery and PNC was lower, 51% and 46%, respectively. The average quality score of 4^+^ ANC visits, institutional delivery and first PNC visit was 0.73, 0.51, and 0.46, respectively. The ANC interventions’ average coverage was higher than 4^+^ ANC visits, The quality-adjusted coverage at the national level, applying the formulas presented in Table 1, was 52%, 33% and 23% for 4^+^ ANC visits, institutional delivery and PNC visit, respectively**.**

**Table 2:** Contact coverage and intervention-specific coverage of different routine MNH visits in Nepal, 2016

A	Contact coverage of MNH visits	Frequency	Yes (%)
	At least four or more ANC visits (N = 1978)	1401	71
	Institutional delivery (N = 1978)	1270	64
	Mothers and newborns received at least one PNC visit within 48 h of childbirth (N = 1978)	999	51

B	Intervention-specific coverage of MNH visits (N = 1978)		
a.	*Antenatal care interventions*		
1.	ANC as per protocol	1078	55
2.	Iron taken	1819	92
3.	Iron taken for at least 180 days	810	41
4.	Told to look for possible problems during pregnancy	1507	76
5.	Told where to go if any pregnancy problems	1517	77
6.	Told to get a postnatal check-up	1086	55
7.	Advice for skilled birth attendance delivery	1419	72
8.	Advice for health facility delivery	1536	78
9.	ANC at health facilities	1751	89
10.	ANC by skilled providers	1703	86
11.	Full neonatal tetanus protection	1801	91
12.	Albendazole is taken during pregnancy	1426	72
13.	Blood pressure measured in ANC visits	1761	89
14.	Urine test in pregnancy	1483	75
15.	Blood test in pregnancy	1319	67
16.	Prepared at least four birth preparedness items out of seven	786	40
17.	Average coverage of all interventions of 4 ANC visits	73% (0.73 out of 1)	
b.	*Institutional delivery interventions (N = 1978)*		
18.	Delivery by skilled health attendants	1277	65
19.	Received cash incentive	912	46
20.	Injection oxytocin labour and after delivery	1021	52
21.	Counselling for injection oxytocin labour and after delivery	568	29
22.	Discharged after 12 h of health facility delivery	790	40
23.	Dried before placenta out	1167	59
24.	Wrapped before placenta out	1167	59
25.	Skin to skin contact immediately	1070	54
26.	Average coverage of all institutional delivery interventions	51% (0.51 out of 1)	
c.	*Postnatal care interventions (N = 1978)*		
27.	PNC of mothers within 2 days	1123	57
28.	PNC of newborns within 2 days	1123	57
29.	PNC newborns by trained health workers	1088	55
30.	PNC of newborns at health facilities	1042	53
31.	Examined cord care	803	41
32.	Measured temperature	775	39
33.	Counselling newborn danger signs	624	32
34.	Counselled on breastfeeding	905	46
35.	Observed breastfeeding	868	44
36.	Observed for dangerous signs	542	27
37.	Bathing 24 h after birth	888	45
38.	Average coverage of all PNC interventions	46% (0.46 out of 1)	

Denominator for each intervention was women who had at least one live birth 2 years prior to the survey, i.e., 1978 women

### Social determinants associated with access to optimal quality 4^+^ ANC visits

Table 3 shows the descriptive characteristics of the quality of routine ANC visits. Among the 1401 women who completed 4^+^ ANC visits, 64% received optimal quality (women with quality scores > 0.8) of 4^+^ ANC visits and rest were with poor quality (quality score > 0 and ≤ 0.8). Women were more likely to receive optimal quality 4^+^ ANC visits if they were a Nepali native speaker (76%), belonged to an advantaged ethnicity (80%), had a job (76%), lived in province seven (79%), had access to a bank account (77%), or perceived a problem of not having female providers (80%) compared with their reference groups (Table 3).

**Table 3:** Social determinants of access to quality 4^+^ ANC visits in Nepal, 2016 (N = 1401)

Determinants	Categories	Frequency (n = 1401)	%	p	4^+^ ANC visits Crude OR (95% CI)	4^+^ ANC visits Adjusted OR (95% CI)
*Structural*						
Wealth rank	Lower	539	61.5	0.242	1.00	
	Upper	862	65.6		1.19 (0.89,1.60)	
Ethnicity	Disadvantaged	903	55.2	< 0.001	1.00	1.00
	Advantaged	498	80.1		3.26 (2.39, 4.45)***	1.68 (1.16, 2.43)**
Religion	Others	186	59.3	0.333	1.00	
	Hindu	1215	64.8		1. 26 (0.79–2.02)	
Occupation	Not working	622	58.8	0.003	1.00	
	Agriculture	596	65.7		1.35 (1.03,1.77)*	
	Working paid	183	76.4		2.27 (1.38, 3.74)**	
Maternal education	Illiterate	305	47.9	< 0.001	1.00	
	Primary	249	58.7		1.55 (1.03, 2.34)*	
	Secondary higher	847	71.4		2.72 (1.92, 3.86)***	
Decision-making	Yes	486	70.7	0.003	1.00	
	No	915	60.5		0.63 (0.47, 0.85)**	
*Intermediary*						
Language	Nepali	673	76.2	< 0.001	1.00	1.00
	Maithili	244	31.7		0.14 (0.10, 0.22)***	0.42 (0.20, 0.88) *
	Bhojpuri	121	55.5		0.39 (0.23, 0.64)***	1.01 (0.48, 2.14)
	Others	363	65.9		0.60 (0.42, 0.87)**	0.82 (0.53, 1.25)
Residence	Urban	802	68.4	0.009	1.00	
	Rural	599	58.1		0.64 (0.46, 0.90)**	
Province	One	266	59.7	< 0.001	1.00	1.00
	Two	298	36.9		0.39 (0.24, 0.65)***	0.74 (0.37,1.49)
	Bagmati	236	76.4		2.18 (1.31, 3.63)**	1.65 (0.97, 2.80)
	Gandaki	124	78.1		2.41 (1.36, 4.28)**	1.69 (1.02, 2.79)*
	Lumbini	272	71.9		1.72 (1.02, 2.92)*	1.47 (0.88, 2.46)
	Karnali	66	68.7		1.48 (0.86, 2.56)	1.37 (0.77, 2.43)
	Sudurpaschim	139	79.2		2.56 (1.57, 4.17)***	2.41 (1.46, 3.97)***
Region	Mountain	94	62.4	< 0.01	1.00	
	Hill	579	74.8		0.56 (0.30, 1.06)	
	Terai	728	55.7		0.42 (0.31, 0.58)***	
Maternal age (years)	15–19	216	54.5	0.003	1.00	
	20–34	1120	66.4		1.65 (1.24, 2.21)***	
	35 +	66	54.9		1.02 (0.55, 1.88)	
Birth order	< 4	1260	66.0	< 0.001	1.00	1.00
	≥ 4	142	46.6		0.45 (0.31, 0.65)***	0.54 (0.35, 0.85) **
Sex of child (index child)	Male	756	65.3	0.337	1.00	
	Female	645	62.5		0.89 (0.69, 1.13)	
Access to bank account	No	904	57.1	< 0.001	1.00	1.00
	Yes	498	76.6		2.47 (1.83, 3.32)***	1.48 (1.08, 2.03) *
Media exposure	Yes	855	69.4	< 0.001	1.00	
	No	546	55.6		0.55 (0.42, 0.73)***	
Last birth (index child)	Wanted	1149	64.9	0.229	1.00	
	Unwanted	252	60.1		0.81 (0.58, 1.14)	
Distance to health facilities as a perceived problem	No problem	588	71.6	< 0.001	1.00	
	Problem	814	58.6		0.56 (0.42, 0.75)***	
*Health system*						
Perceived problem not having female providers	No problem	438	79.6	< 0.001		
	Problem	963	57.0		0.34 (0.24, 0.48)***	
Awareness of health mothers' group in the community	Yes	497	71.6	< 0.001	1.00	
	No	904	59.9		0.59 (0.44, 0.80)***	
C-section delivery	Yes	497	71.6	0.023	1.00	
	No	1235	62.8		0.62 (0.41, 0.94)*	

Significance at ***p < 0.001, **p < 0.01, *p < 0.05. Hosmer Lemeshow test for the model fitness (p = 0.575)

In the multivariable logistic regression, six variables were significantly associated optimal quality 4^+^ ANC visits. Maithili speakers had lower odds (aOR = 0.42; 95% CI 0.20, 0.88) of optimal-quality 4^+^ ANC visits. There were higher odds of receiving optimal quality 4^+^ ANC visits if women belonged to an advantaged ethnicity (aOR = 1.68; 95% CI 1.16, 2.43), and had a bank account (aOR = 1.48; 95% CI 1.08, 2.03) compared to their reference counterparts. Women with high birth order (≥ 4) (aOR = 0.54; 95% CI 0.35, 0.85), and the perceived problem of not having female providers (aOR = 0.47; 95% CI 0.33, 0.67) had lower odds of receiving optimal quality 4^+^ ANC visits than their reference counterparts (Table 3).

### Social determinants associated with access to optimal quality institutional delivery

Among 1270 women who gave birth at a health facility, 43% received optimal quality (women with quality scores > 0.8) institutional delivery. Women who were Nepali speakers (47%), belonged to an advantaged ethnicity (47%), lived in Gandaki province (51%) or a hill region (47%), or who had a history of uptake of 4^+^ ANC visits (51%) had optimal quality of institutional delivery. The multivariable analysis found women who had delivered via C-section had lower odds (aOR = 0.54; 95% CI 0.37, 0.80) of optimal quality of institutional delivery services than women who had a normal vaginal delivery. Women with 4^+^ ANC visits during pregnancy had higher odds (aOR = 1.78; 95% CI 1.33, 2.38) of optimal quality of institutional delivery than women who did not (Table 4).

**Table 4:** Social determinants of access to quality institutional delivery in Nepal, 2016 (N = 1270)

Determinants	Categories	Frequency (N = 1270)	%	p	Institutional delivery cOR (95% CI)	Institutional delivery aOR (95% CI)
*Structural*						
Wealth rank	Lower	405	42.1	0.692	0.95 (0.72, 1.24)	
	Upper	865	43.4		1.00	
Ethnicity	Disadvantaged	819	41.0	0.078	0.79 (0.61, 1.03)	
	Advantaged	451	46.6		1.00	
Religion	Others	193	45.2	0.591	1.00	
	Hindu	1,076	42.6		0.90 (0.62, 1.32)	
Occupation	Not working	636	41.5	0.082	1.00 (0.76, 1.30)	
	Agriculture	460	41.6		1.00	
	Working paid	174	51.9		1.51 (1.00, 2.29)*	
Maternal education	Illiterate	270	38.5	0.278	0.76 (0.51, 1.15)	
	Primary	208	40.5		1.00	
	Secondary higher	792	45.2		0.83 (0.58, 1.17)	
Decision-making	Yes	439	49.3	0.013	1.00	
	No	830	39.7		0.68 (0.50, 0.92)*	
*Intermediary*						
Language	Nepali	589	47.2	0.073	1.00	
	Maithili	204	37.2		0.66 (0.43,1.02)	
	Bhojpuri	141	36.5		0.64 (0.47, 0.89)**	
	Others	336	41.9		0.81 (0.57, 1.14)	
Residence	Urban	781	43.9	0.449	1.00	
	Rural	489	41.6		0.91 (0.70, 1.19)	
Province	One	223	41.0	0.037	1.00	
	Two	280	36.8		0.84 (0.52, 1.35)	
	Bagmati	226	50.6		1.47 (0.89, 2.45)	
	Gandaki	121	50.4		1.47 (0.85, 2.53)	
	Lumbini	239	46.0		1.23 (0.79, 1.91)	
	Karnali	53	37.7		0.87 (0.49, 1.55)	
	Sudurpaschim	128	36.2		0.82 (0.50, 1.33)	
Region	Mountain	59	30.0	0.015	1.00	
	Hill	514	47.1		0.48 (0.33, 0.71)***	
	Terai	697	41.1		0.78 (0.60, 1.03)	
Maternal age (years)	15–19	206	41.2	0.322	1.00	
	20–34	1007	43.9		1.12 (0.78, 1.61)	
	35+	57	32.3		0.68 (0.32, 1.43)	
Birth order	< 4	1153	44.0	0.081	1.00	
	≥ 4	117	33.4		0.64 (0.39, 1.06)	
Sex of child (index child)	Male	698	44.3	0.330	1.00	
	Female	572	41.3		0.88 (0.69, 1.13)	
Access to bank account	No	798	40.9	0.120	0.80 (0.60, 1.06)	
	Yes	472	46.5		1.00	
Media exposure	Yes	807	44.4	0.184	1.00	
	No	463	40.6		0.86 (0.68, 1.08)	
Last birth (index child)	Wanted	1027	43.4	0.678	1.00	
	Unwanted	243	41.3		0.92 (0.62, 1.36)	
Distance to health facilities as a perceived problem	No problem	581	45.3	0.174	1.00	
	Problem	689	41.0		0.84 (0.65, 1.08)	
*Health system*						
Perceived problem not having female providers	No problem	419	47.2	0.057	1.00	
	problem	851	40.9		0.77 (0.59, 1.01)	
Awareness of health mothers’ group in the community	Yes	426	44.2	0.562	1.00	
	No	844	42.4		0.93 (0.72, 1.19)	
C-section delivery	Yes	198	34.0		1.00	0.54 (0.37, 0.80)**
	No	1072	44.7	0.016	1.57 (1.09, 2.26)*	1.00
Quality of 4^+^ ANC visits	Poor	547	33.2	< 0.001	1.00	1.00
	Optimal	723	50.4		2.05 (1.60, 2.63)***	1.78 (1.33, 2.38)***

Significance at ***p < 0.001, **p < 0.01, *p < 0.05. Hosmer Lemeshow test (p = 0.879)

### Social determinants associated with access to optimal quality PNC visit

Among women who completed at least one PNC visit within 48 h after childbirth (n = 999), two-thirds (66.5%) of women who accessed PNC services received optimal quality (women with quality scores > 0.8) PNC visit. Women received optimal quality PNC visits if they had higher wealth status (69%), higher (high school and above) maternal education (70%) or a job (77%) or lived in Gandaki province (76%), had access to a bank account (72%), or delivered via C-section (87%). Multivariable analysis revealed that there was lower odds of receiving optimal quality PNC visits if they were Nepali speakers (aOR = 0.65; 95% CI 0.43, 0.99), from Karnali province (aOR = 0.34; 95% CI 0.18, 0.64) and province Sudurpaschim (aOR = 0.56; 95% CI 0.31, 0.99), and had perceived problems if not having female providers (aOR = 0.67; 95% CI 0.47, 0.96), or high birth order (≥ 4) (aOR = 0.52; 95% CI 0.29, 0.93) compared to their reference groups (Table 5). Women receiving C-sections had higher odds (aOR = 4.20; 95% CI 2.29, 7.68) of receiving optimal quality PNC visits compared to women who had a normal delivery. Similarly, women who received optimal quality 4^+^ ANC visits had higher odds (aOR = 1.69; 95% CI 1.18, 2.43) of optimal quality PNC visits compared to their reference group (Table 5).

**Table 5:** Social determinants of access to quality PNC visits in Nepal, 2016 (N = 999)

Determinants	Categories	Frequency (N = 999)	%	p	PNC visit cOR (95% CI)	PNC visit aOR (95% CI)
*Structural*						
Wealth rank	Lower (40%)	329	60.8	0.035	1.00	
	Upper (60%)	670	69.3		1.45 (1.03, 2.06)*	
Ethnicity	Disadvantaged	615	64.0	0.110	1.00	
	Advantaged	384	70.5		1.35 (0.93, 1.94)	
Religion	Others	145	66.4	0.990	1.00	
	Hindu	854	66.5		1.00 (0.65, 1.55)	
Occupation	Not working	467	63.9	0.060	1.00	
	Agriculture	387	65.6		1.08 (0.77, 1.50)	
	Working paid	146	77.1		1.90 (1.09, 3.32)*	
Maternal education	Illiterate	207	60.9	0.060	1.00	
	Primary	160	61.6		1.03 (0.64, 1.65)	
	Secondary higher	632	69.6		1.47 (0.99, 2.19)	
Decision-making	Yes	354	68.9	0.342	1.18 (0.84, 1.66)	
	No	645	65.2		1.00	
*Intermediary*						
Language	Nepali	484	68.1	0.008	0.80 (0.55, 1.18)	0.65* (0.43, 0.99)
	Maithili	147	56.1		0.48 (0.29, 0.80)**	0.48 (0.22, 1.09)
	Bhojpuri	90	55.4		0.46 (0.26, 0.84)*	0.59 (0.29, 1.22)
	Others	278	72.7		1.00	1.00
Residence	Urban	611	67.3	0.597		
	Rural	388	65.3		0.91 (0.65, 1.28)	
Province	One	184	75.6	0.001	1.00	
	Two	196	53.9		0.38 (0.23, 0.63)***	0.73 (0.37, 1.46)
	Bagmati	196	70.4		0.77 (0.41, 1.45)	0.65 (0.34, 1.23)
	Gandaki	105	76.0		1.03 (0.52, 2.01)	0.92 (0.47, 1.81)
	Lumbini	187	67.4		0.67 (0.41, 1.08)	0.72 (0.44, 1.18)
	Karnali	44	45.1		0.27 (0.15, 0.47)***	0.34 (0.18, 0.64)***
	Sudurpaschim	87	64.3		0.58 (0.36, 0.93)*	0.56 (0.31, 0.99)*
Region	Mountain	60	72.5	0.019	1.05 (0.54, 2.05)	
	Hill	434	71.5		1.00	
	Terai	505	61.5		0.64(0.44, 0.91) *	
Maternal age (years)	15–19	145	65.8	0.838	1.00	
	20–34	813	66.4		1.03(0.69, 1.53)	
	35+	41	71.0		1.27 (0.53, 3.02)	
Birth order	< 4	903	68.4	0.001	1.00	1.00
	≥ 4	96	48.7		0.44 (0.27, 0.71)***	0.52 (0.29, 0.93)*
Sex of child (index child)	Male	563	67.3	0.631	1.00	
	Female	436	65.5		0.92 (0.67, 1.28)	
Access to bank account	No	606	63.3	0.022	1.00	
	Yes	393	71.5		1.45 (1.05, 2.01)*	
Media exposure	Yes	667	70.4		1.69 (1.21, 2.34)**	
	No	606	63.3	0.022	1.00	
Last birth (index child)	Wanted	816	66.0		1.00	
	Unwanted	183	68.7	0.516	0.88 (0.61, 1.28)	
Distance to health facilities as a perceived problem	No problem	468	70.6	0.037	1.00	
	Problem	531	62.9		0.71 (0.51, 0.98)*	
*Health system*						
Perceived problem not having female providers	No perceived problem	349	73.8	0.002	1.00	1.00
	Perceived problem	650	62.6		0.59 (0.42, 0.83)**	0.67 (0.47, 0.96)*
Awareness of health mothers’ group in the community	Yes	364	65.6	0.701	0.94 (0.68, 1.29)	
	No	635	67.0		1.00	
C-section delivery	Yes	165	86.9	< 0.001	4.00 (2.30, 6.95)***	4.20 (2.29, 7.68)***
	No	834	62.4		1.00	1.00
Quality of 4^+^ ANC visits	Poor	383	56.9	< 0.001	1.00	1.00
	Optimal	615	72.5		2.00 (1.45, 2.76)***	1.69 (1.18, 2.43)**
Quality of ID	Poor	541	64.0	0.111		
	Optimal	457	69.4			

*ID* institutional delivery

Significance at ***p < 0.001, **p < 0.01, *p < 0.05. Hosmer Lemeshow test (p = 0.493)

## Discussion

The current analysis of the health service coverage cascade of MNH services in Nepal showed a declining trend for all forms of services throughout pregnancy and the postnatal period. This study also revealed that the privileged women (e.g., advantaged ethnicities or who had bank access) received optimal quality 4^+^ ANC visits while women speaking other than Nepali or with multiple births or living in remote provinces (e.g., Karnali) received poor quality health services during antenatal, childbirth and postnatal period. Women who received optimal quality care in antenatal period than they received optimal quality care in subsequent period such as childbirth or postnatal care visits. Women received poor quality PNC visits if they perceived not having female providers at health facilities was important.

### Service coverage cascade of antenatal, institutional delivery, and postnatal care services

The relatively moderate contact coverage but declining trends of intervention or quality-adjusted coverage suggests women are reaching health facilities but not receiving all essential MNH interventions in their routine MNH visits. Previous studies in Nepal reported poor quality ANC services [65] and low uptake of recommended ANC interventions (e.g., ANC counselling or iron tablets or tetanus toxoid immunisation) [71]. A multi-country analysis from LMICs data revealed low quality of facility delivery [72], 4^+^ ANC visits and PNC visits [73] despite high service contact of respective MNH visits. Studies have also reported poor quality coverage of other health services, such as treatment of sick children (e.g., treatment of diarrhoea, pneumonia) [21] and family planning [18, 21]. Evidence suggests access to health services alone cannot achieve the intended maternal and newborn care outcomes in LMICs, including in Mexico and India [10, 74]. Similar findings of high contact coverage but declining intervention-specific and quality-adjusted coverage are consistent with studies conducted in Bangladesh [75], Cambodia [76], and other LMICs of South Asia and Sub Saharan Africa [21, 25].

In addition to challenges in reaching health facilities, poor quality of health services may be due to inherent weaknesses with the health system, including the lack of trained health workers, and shortages of essential medicines and equipment, and guidelines/standards for MNH services [41, 77]. Universal coverage of quality MNH services is essential for reducing the NMR and MMR and achieving SDG3 targets [15, 78]. A body of literature in Nepal and elsewhere focus on access to health services [31, 41, 79, 80]; however, quality-adjusted coverage, adherence to quality of care standards, and outcome-adjusted coverage are important stages for the effective coverage and intended MNH outcomes [16, 75, 81, 82]. As this study demonstrates, programmatic and policy focus must shift beyond service contact to intervention-specific coverage and quality-adjusted coverage for optimal health outcomes and to achieve the SDG3 targets.

Quality of healthcare is an important determinant of health system performance and health outcomes. Tracking quality of care should therefore be included in tracking progress towards universal health coverage of MNH services [24]. In Nepal, however, as in many other LMICs, monitoring systems have given limited attention to measuring the quality-adjusted coverage of MNH services [83]. This study adopted the measurement of intervention-specific and quality-adjusted coverage, which can be replicated to assess MNH service quality using intervention-specific information available in the HMIS.

### Who was left behind from optimal quality MNH services?

This study identified that women of economic, ethnic, geographic disadvantage, and linguistic minorities (e.g., Maithili speaking women) received poor quality MNH services. These women also have low levels of power and poor social positioning in Nepalese society [84, 85] compared to women from advantaged ethnic groups, who usually also have wealth status. These latter groups are usually Nepali native speakers with good access to education, employment, and health information, compared to disadvantaged ethnicities who belong to the lower strata in the hierarchical caste system of Nepal [86]. Health policy and programs should focus on disadvantaged ethnicities and design context-specific strategies to provide quality care to those groups to address this equity gap.

Women of Karnali province had poor quality MNH services, which is likely to be attributed to scattered settlements, limited road infrastructure and poor access to transport to reach health facilities [58, 87, 88]. Improving these women’s access to care also requires interventions, such as constructing health facilities in more accessible locations and strengthening the road infrastructure. Non-native Nepali speaking women, such as Maithili speaking women, often have low levels of literacy, which might also contribute to poor quality of care, suggesting the need for health education and information programs in local languages. Efforts should also be made to develop a local health workforce to increase the uptake of MNH interventions. Women with high birth order (four births or more) had poor access to quality MNH services during their most recent pregnancy and childbirth. If women have more children, they may not prioritise their latest pregnancy or accept recommended MNH interventions [89]. Uptake of effective family planning services could reduce the number of births [89, 90] where pregnancies are unplanned. Some of the intermediate structural factors that affect access to quality of care sit outside the health sector and require longer-term socioeconomic and developmental interventions. Thus, these contextual interventions need to be targeted among socioeconomically disadvantaged groups to address the social determinants of health, such as women with poor wealth status living Karnali province and ethnic disadvantages, and women speaking Maithili as their first language in Madhesh province. The Reaching the Unraced Strategy (2016–2030) of Nepal has outlined context specific strategies to achieve the universal health coverage by 2030 [91]. In the context of federal health system of Nepal, provincial and local governments have budgets and authorities [92]. These governments can implement focused programs for the most disadvantaged populations to improve the access and quality of health services. Such focussed programs include building maternity waiting home or building health facilities in strategic locations to improve the access and supply of services [48].

### How can be the quality of MNH services modified?

This study revealed important findings that could potentially support the provision and utilisation of optimal quality MNH services in Nepal. First, improving the quality of care in earlier MNH visits (e.g., ANC visits) can ensure optimal quality services in subsequent MNH visits (e.g., institutional delivery or PNC). This finding resonates with existing literature that posits improved quality of care is likely to increase subsequent utilisation of MNH visits [71]; for instance, if women receive optimal quality ANC, then they are more likely to receive optimal quality institutional delivery services or PNC visits [41]. Supply-side interventions include equipping health facilities with adequate supplies, trained health workers (e.g., local health workers who can understand local language and culture) and respectful maternity care, and improved awareness of the uptake of quality pregnancy, childbirth, and PNC services among health facilities.

Second, some women prefer female health providers at MNH services in Nepal. Female health providers can understand the needs of women, especially health issues of reproductive, pregnancy and childbirth [93]. In Nepal, skilled birth attendants (auxiliary nurse midwives with two months of midwifery training or staff nurses) are female cadres and primary care providers of routine MNH services. However, most skilled birth attendants have inadequate skills to identify high-risk pregnancies and complication management during childbirth and the immediate postnatal period. Ensuring provision of trained and skilled birth attendants in health facilities, mostly in rural health facilities could be an interim strategy, while production and recruitment of graduate midwives could fill the gaps of midwifery care in Nepal.

Third, women delivering by C-section received poor quality institutional delivery but optimal-quality PNC visits. This may be because these women usually have a longer hospital stay and are thoroughly examined before discharge. Nepal has higher rates of C-section delivery than recommended by the WHO (5*–*15%), especially in private hospitals where the C-section rate is up to 80% [94] and there are limited midwives trained to assist with C-section procedures [95–97]. Further research is warranted to understand the quality of routine intrapartum care and essential newborn care for mothers delivering via C-section and their newborns.

## Implications of the study

This study has some policy and methodological implications. In the past decade, access to health services has been increasing, but MNH outcomes remain poor and inequitable. This study revealed that poor MNH services were received by socioeconomically marginalised groups or those living in remote and peripheral areas. Thus, health system efforts need to focus beyond contact coverage and improve quality-adjusted coverage. Improving access to quality care also requires multisectoral actions to address the social determinants of health. Additionally, local governments can strengthen municipal health systems by ensuring trained health workers, essential medicine and supplies for the uptake of quality health services, with tailored interventions to address the needs of most marginalised populations and ensure quality health services for improved health outcomes.

Measurement of quality of care requires multiple data sources, such as users, providers, facility inventory, and observations of interactions between providers and users [15, 98]. However, this study demonstrates demographic and health survey data can be used to assess health care quality and identify the population-level coverage of quality health services. This study assessed the quality of routine MNH visits, taking information on services users’ engagement with the health system and using household surveys and data on adequate care, timely care, and frequent and skilled care while women visit health facilities. Particularly, this study examined the population-level health service coverage cascade, especially intervention-specific and quality-adjusted coverage of MNH services. The approach used in this study can be replicated to estimate the population-level coverage of primary health care services, including family planning, child health and nutritional health services, especially in those countries with limited facility-level data. Finally, this is the first study from Nepal that applied Marshall and colleagues’ approach of measurement of effective coverage using health service coverage cascades of MNH services [16]. Therefore, this study could be a reference for researchers to assess the health services coverage cascades of other services (e.g., infectious diseases, non-communicable diseases) using demographic and health survey data at national and subnational levels of other countries.

## Limitations of the study

This study has a few limitations. First, outcome variables were self-reported by women during face-to-face interviews, which may have led to recall bias and social desirability bias. However, this study restricted analysis among women who had a live birth 2 years before the survey (2014–2016), which is a relatively shorter recall period than other published studies using NDHS datasets. Second, we used secondary data and were limited to using available variables in the dataset of the NDHS 2016. We referenced national medical standards for the MNH of Nepal [61], including maternal and newborn care items, and calculated the quality of care of MNH visits. Third, while calculating the quality score, each service item was given equal weightage based on previous literature in the field [21, 64]. These interventions could have a varied impact on the survival of mothers and babies. We also took references of previous studies conducted in other counties, which gave equal weightage for interventions to calculate the quality score [18, 23, 64, 75], which we believe is optimal than having no estimates. Future studies can be conducted by giving proportionate weightage according to their impact on pregnancy and babies. Finally, linear regression of the quality scores was not possible due to their distribution. Therefore, we dichotomised the quality scores. Although there is no gold standard of cut-off points for categorizing poor- or optimal-quality care, literature suggests that a higher cut-off point is quality categorisation. We referenced previous studies undertaken in Kenya [64] and Nepal [65] and dichotomised the quality score to identify the social determinants of optimal quality health care.

## Conclusions

Despite improved access to contact coverage of routine MNH services, there was low intervention-specific and quality-adjusted coverage. Women with ethnic disadvantage and living in remote areas received poor quality MNH services. This study demonstrated that health service coverage cascade, especially quality-adjusted coverage can be examined using data from representative household surveys. We recommend that the demographic and health surveys collect additional intervention-specific information of routine health services that can be used to estimate the quality of health services at the population level. Supply- and demand-side multisectoral actions are needed to address several social determinants of health affecting poor quality MNH visits in Nepal. Supply-side strategies, such as recruiting female health providers and providing health commodities in health facilities, could improve the health system's responses to the delivery of optimal quality MNH services. Modifying the social determinants of health, community engagement, and health awareness through mass media on the antenatal, childbirth, and postnatal period could increase the demand for services for optimal quality routine MNH visits. Multisectoral interventions could improve routine MNH visits, including arranging transportation facilities or constructing local bridges and roads to reach health facilities. Health policies and programs should focus on women with socioeconomic disadvantage living in remote areas.

## Supplementary Information

**Additional file 1: Table S1.** Study variables included in the assessment of quality of MNH visits in Nepal, NDHS 2016. **Table S2.** List of Intervention-specific included to estimate the quality score of MNH visits in Nepal, 2016. **Table S3.** Characteristics of women who had a live birth in the 2 years preceding the survey in Nepal, NDHS 2016.

## Data Availability

Data used in this study are publicly available secondary data obtained from the DHS (https://dhsprogram.com/data/available-datasets.cfm) program.

## Abbreviations

ANC: Antenatal care
4^+^ ANC: Four or more antenatal care
aOR: Adjusted odds ratio
CI: Confidence interval
cOR: Crude odds ratio
C-section: Caesarean section
EC: Effective coverage
EA: Enumeration area
HF: Health facility
HMIS: Health Management Information System
ID: Institutional delivery
LMICs: Low and middle-income countries
MNH: Maternal and newborn care
MMR: Maternal mortality ratio
NMR: Neonatal mortality rate
NDHS: Nepal Demographic and Health Survey
OR: Odds ratio
PNC: Postnatal care
PSU: Primary sampling unit
SBA: Skilled birth attendant
TT: Tetanus toxoid
