# Supplementary material for: High coverage but low quality of maternal and newborn health services in the coverage cascade: who is benefitted and left behind in accessing better quality health services in Nepal?
Source: Reprod Health. 2022 Jul 19;19:163. doi: 10.1186/s12978-022-01465-z (PMC9297647; doi:10.1186/s12978-022-01465-z)
Supplement: Supplementary file 1 — Additional file 1: Table S1. Study variables included in the assessment of quality of MNH visits in Nepal, NDHS 2016. Table S2. List of Intervention-specific included to estimate the quality score of MNH visits in Nepal, 2016. Table S3. Characteristics of women who had a live birth in the 2 years preceding the survey in Nepal, NDHS 2016. [file 12978_2022_1465_MOESM1_ESM.doc]

Supplimentaty file Table S1: Study variables included in the assessment of effective coverage of MNH visits in Nepal, NDHS 2016

| **Variables** | **Categories** | **Descriptions** |
| --- | --- | --- |
| **Independent variables** |  |  |
| **Structural** |  |  |
| Wealth status | Lower wealth status (lower 40%); Upper wealth status (upper 60%) | NDHS data had a variable wealth quintile, which was calculated based on scores generated from principal components analysis of households' assets (40 items). It was grouped into two categories: lower (poorest, poor, or lower two quintiles); and upper (middle, richer, and richest or upper threequintiles) wealth status. |
| Ethnicity | Disadvantaged; Advantaged | Disadvantaged: Dalit, Muslims, and Terai caste, Janajatis disadvantaged) and advantaged: Brahmin/Chhetri, advantaged Janajatis). |
| Maternal education | Illiterate; Primary; Secondary or higher | Illiterate: Cannot read and write; Primary: 1-8 grade.  Secondary or higher: 9^th^ grade and higher |
| Religion | Others; Hindu | Others include Muslims, Christian, Buddhist |
| Maternal occupation | Not working (housewife); Agriculture; Working paid | Based on the response of respondents, not working women are housewives (usually husbands of such women have paid jobs), agriculture (family's main source of income is agriculture), and paid job |
| Decision-making authority | No; Yes | Whether women participated in at least one of the decisions regarding their health care, purchases or visits to their family or relatives' households |
| **Intermediary** |  |  |
| Languages | Nepali, Maithili, Bhojpuri, and Others (e.g., Tharu, Newari) | The primary language of the respondents |
| Maternal age (in years) | 15-19, 20-34, 35 and above | 15-19, and 35+ years are more at-risk groups |
| Residence | Urban; Rural | Municipalities are called urban, and remaining parts are called rural areas. This rural-urbancategorizationis based on socioeconomic indicators of the population. Municipalities have a higher populationand developmentindicators. However, many municipalities which are considered as urban areas do not have adequate development facilities |
| Provinces | One, Madhesh, Bagmati, Gandaki, Lumbini, Karnali, Sudurpaschim | Province 1 is yet to be named by provincial assembly |
| Region | Mountain; Hills; Terai | Ecological region |
| Birth order | <4; ≥4 | Numbers of children in the family. |
| Sex of index child | Male; Female | Sex of last birth child |
| Access to bank account | No; Yes | This is a marker of financial empowerment and access to finance |
| Media exposure | No; Yes | Received health related message from at least one of the following once a week: newspaper, radio, or television |
| Last birth (index child) | Unwanted; Wanted | Women perceived the youngest child intentional or not |
| Distance to HFs is a perceived problem | No problem; a big problem | Perception of problem associated with distance to HF for medical care |
| **Health system** |  |  |
| Perceived problem not having female providers | No problem, big problem | Perceived problem, or not; if no female health provider for healthcare |
| Awareness on health mothers' group | No; Yes | Awareness of health mothers' groups in the respective wards |
| Mode of delivery | Normal; C-section | Types of childbirth services received by women in health facility |
| Quality of 4^+^ANC | Poor; optimal | If coverage score >0 and ≤0.8=0 (poor quality); if score >0.8=1 (optimal quality) |
| Quality of institutional delivery | Poor; optimal | If coverage score >0 and ≤0.8=0 (poor quality); if score >0.8=1 (optimal quality) |
| **Outcome variables** |  |  |
| Quality of 4^+^ANC visits | Poor; optimal | If coverage score >0 and ≤0.8=0 (poor quality); if score >0.8=1 (optimal quality) |
| Quality of institutional delivery | Poor; optimal | If coverage score >0 and ≤0.8=0 (poor quality); if score >0.8=1 (optimal quality) |
| Quality of PNC visit | Poor; optimal | If coverage score >0 and ≤0.8=0 (poor quality); if score >0.8=1 (optimal quality) |

Table S2: list of Intervention specific included to estimate the quality score of MNH visits in Nepal, 2016

| **A.** | **Antenatal care interventions (N=1978)** |
| --- | --- |
|  | ANC as per protocol |
|  | Iron taken |
|  | Iron taken for at least 180 days |
|  | Told to look for possible problems on pregnancies |
|  | Told where to go if any problems |
|  | Told to get a postnatal checkup |
|  | Advice for skilled birth attendance delivery |
|  | Advice for health facility delivery |
|  | ANC at health facilities |
|  | ANC by skilled providers |
|  | Full neonatal tetanus protection |
|  | Albendazole is taken during pregnancy |
|  | BP measured in ANC visits |
|  | Urine test in pregnancy |
|  | Blood test in pregnancy |
|  | Prepared at least four birth preparedness items out of seven |
| **B.** | **Institutional delivery interventions (N=1978)** |
|  | Delivery by skilled health personnel |
|  | Received cash incentive |
|  | Injection oxytocin labour and after delivery |
|  | Counselling for injection oxytocin labour and after delivery |
|  | Discharged from HF 12 hours after birth |
|  | Dried before placenta out |
|  | Wrapped before placenta out |
|  | Skin to skin contact immediately |
| **C.** | **Postnatal care interventions (N=1978)** |
|  | PNC of mothers within 2 days |
|  | PNC of newborns within 2 days |
|  | PNC newborns by trained health workers |
|  | PNC of newborns at HFs |
|  | examined cord care |
|  | measured temperature |
|  | counselling newborn danger signs |
|  | counselled on breastfeeding |
|  | observed breastfeeding |
|  | observed for dangerous signs |
|  | Bathing 24 hours after birth |

Table S3: Characteristics of women who had a live birth in the two years preceding the survey in Nepal, NDHS 2016 (N=1978)

| **Determinants** | **Categories** | **Frequency, n (%)** |
| --- | --- | --- |
| **Structural** |  |  |
| Wealth status | Lower (40%) | 832 (42.0) |
|  | Upper (60%) | 1146 (58.0) |
| Ethnicity | Disadvantaged | 1374 (69.5) |
|  | Advantaged | 604 (30.5) |
| Religion | Others | 306 (15.5) |
|  | Hindu | 1672 (84.5) |
| Maternal education | No | 570 (28.8) |
|  | Primary | 391 (19.8) |
|  | Secondary or higher | 1016 (51.4) |
| Maternal occupation | Not working | 928 (46.9) |
|  | Agriculture | 824 (41.6) |
|  | Working paid | 227 (11.5) |
| Perceived violence | No | 1397 (70.6) |
|  | Yes | 581 (29.4) |
| Decision-making authority | No | 1324 (66.9) |
|  | Yes | 654 (33.0) |
| Household head | Male | 1438 (72.7) |
|  | Female | 540 (27.3) |
| **Intermediary** |  |  |
| Language | Nepali | 839 (42.4) |
|  | Maithili | 360 (18.2) |
|  | Bhojpuri | 267 (13.5) |
|  | Others | 512 (25.9) |
| Residence | Urban | 1062 (53.7) |
|  | Rural | 916 (46.3) |
| Provinces | One | 338 (17.1) |
|  | Two | 513 (25.9) |
|  | Bagmati | 312 (15.8) |
|  | Gandaki | 164 (8.3) |
|  | Lumbini | 364 (18.4) |
|  | Karnali | 121 (6.1) |
|  | Sudurpaschim | 166 (8.4) |
| Region | Mountain | 131 (6.6) |
|  | Hills | 760 (38.4) |
|  | Terai | 1087 (55.0) |
| Maternal age (in years) | 15-19 | 291 (14.7) |
|  | 20-34 | 1570 (79.7) |
|  | 35+ | 106 (5.3) |
| Birth order (index child) | <4 | 1678 (84.8) |
|  | ≥4 | 300 (15.2) |
| Sex of index child | Male | 1063 (53.7) |
|  | Female | 915 (46.2) |
| Access to bank account | No | 1367 (69.1) |
|  | Yes | 611 (30.9) |
| Media exposure | No | 911 (46.0) |
|  | Yes | 1067 (54.0) |
| Last birth (index child) | Unwanted | 418 (21.1) |
|  | Wanted | 1560 (78.8) |
| Distance to health facilities as a perceived problem | No problem | 763 (38.6) |
|  | Big problem | 1213 (61.4) |
| **Health system** |  |  |
| Perceived problem not having female providers | No problem | 562 (28.4) |
|  | Big problem | 1416 (71.6) |
| Awareness on health mothers' group in the community | No | 1340 (67.7) |
|  | Yes | 638 (32.3) |
| Mode of delivery | Normal (veginal) | 1780 (90.0) |
|  | C-section | 198 (10.0) |
| Quality adjusted coverage of 4^+^ANC visits (n=1401) | Poor quality | 504(36.0) |
|  | optimal quality | 897(64.0) |
| Quality adjusted coverage of institutional delivery (n=1270) | Poor quality | 724 (57.0) |
|  | optimal quality | 546(43.0) |
| Quality adjusted coverage first PNC visit (n=999) | Poor quality | 335 (33.5) |
|  | optimal quality | 664 (66.5) |
